# Mechanical Properties, Thermal Stability, and Formaldehyde Emission Analysis of Nanocellulose-Reinforced Urea–Formaldehyde Resin and Its Mechanism

**DOI:** 10.3390/polym17101402

**Published:** 2025-05-20

**Authors:** Xue Deng, Zhu Liu, Zhongwei Wang, Zhigang Wu, Dan Li, Shoulu Yang, Shiqiang He, Ning Ji

**Affiliations:** 1 Guizhou Academy of Forestry, Guiyang 550002, China; xue1208776758@163.com (X.D.); liuzhu9206@126.com (Z.L.); zhongweiwang202204@163.com (Z.W.); ldan0402@163.com (D.L.); jining420@163.com (N.J.); 2 College of Forestry, Guizhou University, Guiyang 550025, China; wzhigang9@163.com; 3 Guizhou Liping Rocky Desertification Ecosystem Observation and Research Station, Liping 557300, China

**Keywords:** urea–formaldehyde resin, nanocrystalline cellulose, nanofibrillated cellulose

## Abstract

In this research, a urea–formaldehyde (UF) resin was modified with nanocrystalline cellulose (NCC) and nanofibrillated cellulose (CNF), and the properties of the modified resin were comprehensively evaluated by combining the techniques of infrared spectroscopy (FTIR), X-ray diffraction (XRD), differential scanning calorimetry (DSC), thermogravimetric analysis (TGA), and scanning electron microscopy (SEM). The results showed that (1) the introduction of NCC and CNF significantly changed the hydrogen bonding network of the UF resin, in which CNF enhanced the internal hydrogen bonding of the resin through its long-chain structure and elevated the cross-linking density. NCC increased the crystallinity of the resin, while CNF enhanced the overall performance of the resin by improving its dispersion. (2) The composite curing agent system significantly reduced the curing temperature of the resin, resulting in a more homogeneous and efficient curing reaction, and the CNF-modified UF exhibited better thermal stability. (3) The addition of NCC and CNF significantly improved the dry and water-resistant bonding strengths of the resins. In addition, the use of complex curing agent further enhanced the bonding strength, especially in the CNF-modified system; the addition of complex curing agent increased the dry bonding strength to 1.60 MPa, and the water-resistant bonding strength reached 1.13 MPa, which showed a stronger cross-linking network and structural stability. (4) The addition of NCC and CNF led to a significant reduction in the free formaldehyde content of UF resins, resulting in respective levels of 0.17% and 0.14%. For plywood bonded with the CNF-modified UF resin, formaldehyde emissions were measured at 0.35 mg/L, which were markedly lower than the 0.54 mg/L of the unmodified sample. This further highlights CNF’s effectiveness in minimizing formaldehyde release. (5) Overall, CNF is superior to NCC in improving the thermal stability, bonding strength, water resistance, formaldehyde release, and overall performance of the resin. The use of complex curing agents not only optimizes the curing process of the resin but also further enhances the modification effect, especially for CNF-modified resins, which show more significant performance advantages.

## 1. Introduction

Urea–formaldehyde (UF) adhesives have been widely used in the wood industry due to the advantages of a low cost, excellent water solubility, higher curing strength, and low production cost [1]. However, the high reversibility and low stability of the amino-methylene linkage in the UF molecular structure result in poor durability at high temperatures and high humidity, and the preparation of UF-bonded plywood is susceptible to hydrolysis during use and continuously releases formaldehyde, which poses a serious threat to human health [2,3]. There are two main sources of formaldehyde release from UF adhesives: one is the free formaldehyde that is not involved in the reaction; the other is condensed formaldehyde, methylene, and hydroxymethyl [4]. In order to reduce formaldehyde release from UF adhesives, the F/U molar ratio can be reduced, as well as modification using urea, phenol, tannin, and lignin [5,6,7,8,9]. However, most of the above can only temporarily inhibit free formaldehyde, and it is difficult to continuously play the role of inhibiting formaldehyde release.

Nanotechnology, as an emerging frontier technology, is used to change the structure and optimize the properties of target substances by manipulating nanoscale substances [10]. Numerous scholars have devoted themselves to modifying urea–formaldehyde resins using nanomaterials to reduce their formaldehyde emissions, and the commonly used nanomaterials include nanosilica [11], carbon nanotubes [12,13,14], carbon fibers [15,16,17,18], and glass fibers [19,20]. Nanocellulose, as the smallest physical structural unit of cellulose, has many excellent properties, such as excellent mechanical properties, a large surface area, degradability, easy to modify, high strength, and high toughness. Its surface is enriched with a large number of hydroxyl groups, and these hydroxyl groups produce a strong tendency of self-association through the formation of hydrogen bonding, which is capable of generating interaction forces with the UF condensate [17], thus exhibiting the potential advantage of enhancing the strength of the adhesive.

Based on this, research on the use of nanofibrillated cellulose to enhance the properties of adhesives have been continuously attempted [21,22,23]. For example, López-Suevos [24] modified PVAc using nanofibrillated cellulose (CNF) and succeeded in improving its heat resistance. Veigel et al. [25,26] improved the fracture toughness of modified UF by 45% by blending CNF with UF with the addition of 2% CNF. In addition, they blended CNF, UF, and MUF and determined that when CNF was added at 1%, CNF had a significant reinforcing effect on the oriented strand boards, with a 16% increase in strength. A study by Yildirim [27] found that the composite reinforcement of nanofibrillated cellulose with borax significantly increased the modulus of rupture and modulus of elasticity of the plywood by about 26% and the gluing strength by about 47%, while the free formaldehyde content was reduced by 34%. Wibowo [28] indicated that nanofibrillated cellulose effectively improved the curing properties of urea–formaldehyde resins and reduced formaldehyde release. Compared with unmodified urea–formaldehyde resin, the addition of CNF resulted in a shorter gelling time, higher tensile shear strength, and lower formaldehyde release. When the addition of 3% nanofibrillated cellulose was used in modified UF resin-treated plywood, the dry and wet strengths were increased by 30% and 42%. Zhang [29] treated nanocrystalline cellulose (NCC) with a modifier to improve the compatibility between NCC and UF, and the treated NCC was enhanced in terms of thermal stability and crystallinity, the gluing properties of UF adhesives were improved, and formaldehyde release was reduced. Yalçın [30] found that the best performance of particleboard produced by a urea–formaldehyde resin adhesive modified with a 0.5% mass fraction of titanium dioxide nanoparticles and 1% mass fraction of cellulose nanoparticles was found to have a modulus of elasticity of 2570 MPa and a gluing strength of 1.1 MPa. Mesquita [31] blended NCC with UF, and the viscosity of the UF adhesive increased with the increase in NCC additive, when the amount of NCC added was 5%, the viscosity hindered the dispersion of the adhesive in the particleboard, while the best performance of the particleboard was achieved when the amount of NCC added was 1%.

In this research, UF was modified with NCC and CNF, and the properties of the modified resin were comprehensively evaluated by combining infrared spectroscopy (FTIR), X-ray diffraction (XRD), differential scanning calorimetry (DSC), thermogravimetric analysis (TGA), and scanning electron microscopy (SEM). The innovativeness of this research lies in that (1) we conducted a systematic analysis of the distinctions between NCC and CNF in modifying UF resins, uncovering their mechanisms of influence on the resins’ chemical structure, thermal stability, adhesive properties, and formaldehyde emissions. (2) We investigated the part played by nanofibrillar cellulose in the curing of UF resins and revealed its impacts on the thermodynamics of the curing reaction and the formation of cross-linked networks. (3) We optimized the curing behavior of MUF resins by combining them with a complex curing agent system, enhancing their overall performance and exploring the synergistic effects of nanocellulose and curing agents. This research deepens the understanding of nanocellulose’s role in UF resin modification and offers theoretical support for developing low-formaldehyde, high strength, and aging-resistant UF resins. It also provides significant guidance for applying UF resins in wood adhesives, composites, and other high-performance fields.

## 2. Materials and Methods

### 2.1. Materials

Formaldehyde (wt 37%), analytically pure, was obtained from Sinopharm Chemical Reagent Co., Ltd. (Shanghai, China); other reagents such as NaOH, melamine, urea, etc., were analytically pure and purchased from Sinopharm (Guizhou, China); formic acid (wt 88%) was analytically pure and purchased from Tianjin Wind Ship Chemical Reagent Technology Co. (Tianjing, China). Ammonium chloride was analytically pure and obtained from Tianjin Yongda Chemical Co., Ltd. (Tianjing, China); ammonium dihydrogen phosphate was analytically pure and obtained from Shanghai McLean Biochemical Technology Co., Ltd. (Shanghai, China). Nanofibrillated cellulose (CNF) and nanocrystalline cellulose (NCC), both analytically pure, were purchased from Wuhan Kemik Bio-medical Technology Co. (Wuhan, China). Poplar veneer (*Populus* spp., moisture content 8–10%) with a veneer width of 400 mm × 400 mm and thickness of 1.5 mm was purchased from Jiangsu Muyang, China.

### 2.2. Preparation of the UF Resin

In a three-necked flask equipped with a condenser tube and stirring bar, 320 g of formaldehyde solution was added, the first amount of urea (105 g) was added, stirring was initiated to uniformly mix the system, and the pH was adjusted to 7.5–8.0. The temperature was raised to 90 °C within 40–50 min and the reaction was kept at a constant temperature for 30 min. Subsequently, 10 g of melamine (5% of the total mass of urea) was added and the reaction was continued for another 30 min. After cooling down to 70 °C, the pH was adjusted to 5.4–5.6, a second amount of urea (29.2 g) was added, and the reaction was continued with stirring until a white flocculent appeared in the system. Subsequently, the pH was adjusted to 7.4–7.6, the third amount of urea (67.2 g) was added, and the reaction was continued for 30 min. Finally, the pH was adjusted to 8.0–8.5, the reaction was cooled and discharged, and the blank UF resin was obtained.

### 2.3. Preparation of Nanocellulose-Modified UF Resins

In a three-necked flask equipped with a condenser tube and stirring bar, 320 g of formaldehyde solution was added, the first amount of urea (105 g) was added, stirring was initiated to uniformly mix the system, and the pH was adjusted to 7.5–8.0. The temperature was raised to 90 °C within 40–50 min and the reaction was kept at a constant temperature for 30 min. Subsequently, 10 g of melamine (5% of the total mass of urea) was added and the reaction was continued for 30 min. Nanofibers were added at 0.8%, 1.0% and 1.2% (of the total mass of urea), and the reaction was carried out for 30 min, after cooling down to 70 °C. The pH was adjusted to 5.4–5.6, a second amount of urea (29.2 g) was added, and the reaction was stirred continuously until white flocs appeared in the system. Subsequently, the pH was adjusted to 7.4–7.6, a third amount of urea (67.2 g) was added, and the reaction was continued for 30 min. Ultimately, the pH was adjusted to 8.0–8.5, and the reaction was cooled and discharged.

When adding the NCC modification, the adhesives obtained were recorded as UF/NCC_0.8_, UF/NCC_1.0_, and UF/NCC_1.2_ (the subscript number on the right indicates the amount of NCC added based on the total mass of urea). When adding the CNF modification, the adhesives obtained were recorded as UF/CNF_0.8_, UF/CNF_1.0_, and UF/CNF_1.2_ (the subscript number on the right indicates the amount of CNF added based on the total mass of urea). Adhesive solids and viscosity were tested according to the provisions of the national standard GB/T 14074-2017 [32].

### 2.4. Plywood Preparation and Performance Testing

Before pressing the plywood, 2% of the curing agent based on the adhesive solids was added and mixed well. There were two types of curing agents, the single curing agent NH_4_Cl and the compound curing agent (consisting of NH_4_Cl and ammonium dihydrogen phosphate), and the curing agents were directly added to the adhesive. The laboratory made its own three-layer poplar plywood with a double-sided sizing of 180 g/m^2^, and after the sizing was finished, it was left for 2–3 min to assemble it, and then it was hot-pressed. The plywood hot pressing process was a time of 5 min, temperature of 120 °C, and pressure of 1.5 MPa. The plywood boiling water-resistant gluing strength was tested with reference to the test method of Class I plywood in GB/T 17657-2013. The obtained plywood was left in an air-drying environment for 24 h. It was then cut into specimens measuring 100 mm × 25 mm for the gluing performance test. The dry strength and warm water strength were measured in accordance with the national standard GB/T 17657-2013 [33].

### 2.5. Formaldehyde Release Test

Free formaldehyde content test: Take 20 mL of 15 wt% sodium sulfite in a 250 mL conical flask, add 1~2 drops of thymolphthalein, and then add an appropriate amount of sodium hydroxide solution so that it happened to appear slightly blue. Weigh about 5 g (accurate to 0.01) of the urea–formaldehyde resin adhesive sample in another conical flask with an electronic balance, add distilled water and shake to make the urea–formaldehyde resin adhesive sample fully dissolve, add 1~2 drops of thymolphthalein, and then add an appropriate amount of sodium hydroxide solution to make it happen to appear as a slight blue color. Remove 10 mL of the 0.5 mol/L hydrochloric acid solution and add it to the above-dissolved sample of urea–formaldehyde resin adhesive, and quickly add about 10 drops of thymolphthalein with a rubber-tipped burette to the previously prepared sodium sulfite solution. Using a concentration of 0.1 mol/L sodium hydroxide solution as the standard solution, titrate the specimen so that it just appeared slightly blue and did not change color within half a minute, which was recorded as the end point of the titration. The specimen was titrated three times in parallel and the above steps were repeated with 50 mL of distilled water instead of the specimen for a blank experiment.

Plywood formaldehyde emission test: According to the method in the national standard GB/T 17657-2013 [33] “Test Methods for Physical and Chemical Properties of Artificial Boards and Veneered Artificial Boards”, the formaldehyde emissions of plywood were tested by the desiccator method. (1) Collection of formaldehyde. A crystallization dish with a diameter of 240 mm was placed at the bottom of a clean desiccator, about 300 mL of distilled water was added to the crystallization dish, a wire support net was placed above the crystallization dish, and a metal bracket was placed on top of the dish; 10 pc small cut specimens were placed on it such that the specimens did not touch each other, the desiccator was covered with a lid, and the desiccator was placed in a smooth constant temperature box and left at (20 ± 0.5) °C for 24 h. The formaldehyde released from the specimens was absorbed by distilled water, which was the solution to be tested. (2) Determination of formaldehyde release. According to the method of the national standard, the formaldehyde standard liquid was calibrated, the absorbance was measured at 412 nm by a UV spectrophotometer, and the standard curve was plotted so as to determine the formaldehyde content of the liquid to be tested

### 2.6. Infrared Spectroscopy (FT-IR) Analysis

The adhesive was baked at 120 °C for 2 h and then pulverized into powder with a particle size of about 297 μm. Before the test started, the Fourier Transform Infrared (FTIR) Spectrometer was turned on, warmed up for about 20 min, and the specimens were prepared simultaneously. The resin was mixed according to a resin to potassium bromide mass ratio of 1:100, and after being well ground and homogenized, it was pressed under pressure for 30 s to form a transparent or semi-transparent sheet. Subsequently, the sample was placed in the detection chamber, and the scanning range was set to 4000–400 cm^−1^ with a scan time of 32.

### 2.7. Scanning Electron Microscopy (SEM) Tests

Scanning electron microscope (SEM) model S-3400N was used to analyze the cross-sectional morphology of the cured resin. In this test, the resin was cured at room temperature and the cross-section was observed by SEM. During testing, an accelerating voltage of 12.5 kV was selected and the resin was placed with the cross-section facing upwards, followed by gold spraying on the surface to improve electrical conductivity. After the gold spraying process was completed, the samples were placed in a scanning electron microscope, the target area was selected for imaging, and the images were acquired at different magnifications. Ultimately, the curing characteristics and microstructural differences were analyzed by comparing the changes in resin cross-sectional morphology before and after treatment.

### 2.8. Differential Scanning Calorimetry (DSC) Analysis

Differential scanning calorimetry (DSC) was used to analyze the curing performance of the resin. A DSC 204 F1 model manufactured by NETZSCH (Rodgau, Germany) was used for this test. The experiment was carried out under the protective atmosphere of nitrogen (N_2_), and the temperature range of the test was set at 25–250 °C with a temperature increase rate of 10 °C/min. 

### 2.9. Thermal Performance (TG) Analysis

The thermal stability of the resin was determined by a thermogravimetric analyzer (TG). A thermogravimetric analyzer model TG/DTA7300 (Rodgau, Germany) was used for the test. First, the resin was dried at 120 °C for 2 h, then pulverized and sieved through a 40–60 mesh to obtain a homogeneous powder sample. The samples were weighed at 5–7 mg and placed in a high-purity nitrogen (N_2_) atmosphere with the nitrogen flow rate set at 20 mL/min. During the experiment, the samples were heated up from room temperature to 600 °C at a rate of 10 °C/min.

### 2.10. X-Ray Diffraction (XRD) Analysis

The crystalline properties of the resin were characterized by X-ray diffraction (XRD) [34,35]. A TTR XRD type X-ray diffractometer manufactured by Rigaku (Tokyo, Japan) was used for the tests. First, the resins were baked at 120 °C for 2 h and subsequently pulverized and sieved through a 80–100 mesh to ensure the homogeneity of the samples. A Cu target (λ = 0.154060 nm) was selected as the X-ray source, and the scanning range (2θ) was set to 5–80° with a step size of 0.02°, a scanning rate of 6°/min, a tube current of 120 mA, and a tube voltage of 40 kV.

## 3. Results and Discussion

### 3.1. Infrared Spectral Analysis

**Figure 1:**
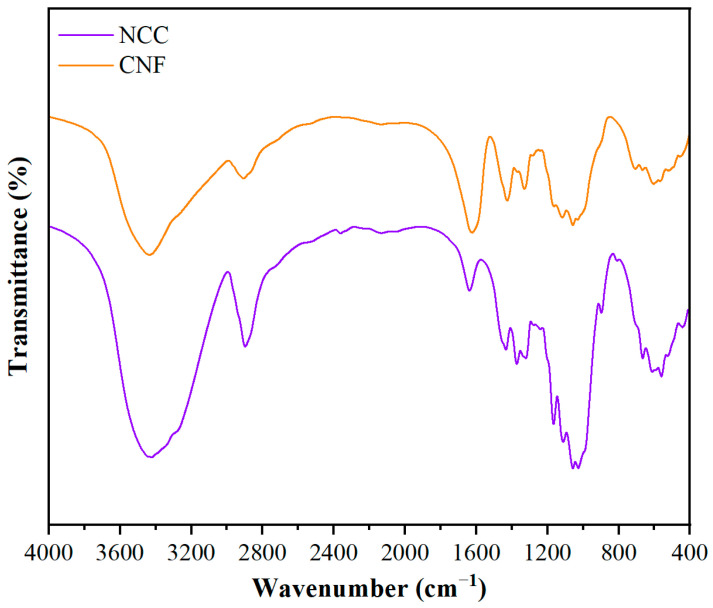
Infrared spectra of two nanofibrillar cellulose preparations: NCC and CNF.

The infrared spectra of two types of nanocellulose, nanocrystalline cellulose (NCC) and nanofibrillated cellulose (CNF), are shown in Figure 1. Both NCC and CNF are cellulose-dominated in their chemical compositions, but the infrared spectra show some differences due to the differences in morphology, degree of crystallinity, and surface chemical properties. In the range of 3200–3400 cm^−1^, both NCC and CNF exhibit broad peaks attributed to the stretching vibration of -OH. However, the -OH absorption peaks of NCC are narrower than those of CNF, indicating stronger hydrogen bonding and higher crystallinity of NCC, whereas CNF has more dispersed intermolecular hydrogen bonding due to its longer fiber structure, resulting in a broader peak shape. In the range of 2800–2900 cm^−1^, both C-H symmetric and asymmetric stretching vibrational peaks are present, but those in NCC is weaker than the peaks in CNF, suggesting that there are fewer C-H groups on its surface, which may be related to the degradation of some of the amorphous regions of NCC during acid hydrolysis. Both NCC and CNF exhibit characteristic absorption peaks typical of C-O-C (1030 cm^−1^) and β-1,4-glucosidic bonds (1055 cm^−1^), but the peak position of NCC is slightly shifted toward higher wavelengths, suggesting a more structurally regular and more crystalline. In addition, the CH_2_ deformation vibration (1430 cm^−1^) and the β-glycosidic bond deformation vibration (897 cm^−1^) show a higher absorption intensity for NCC than for CNF, which further verifies the higher crystallinity of NCC, whereas CNF exhibits a weaker characteristic absorption due to its longer fiber structure and larger amorphous region.

Overall, NCC has higher crystallinity in terms of narrowing of the hydroxyl stretching vibrational peaks, weakening of the C-H stretching peaks, and enhancement of the β-glycosidic bonds. CNF, on the other hand, has more amorphous regions and exhibits broader hydroxyl vibrational peaks and stronger C-H bond absorption.

**Figure 2:**
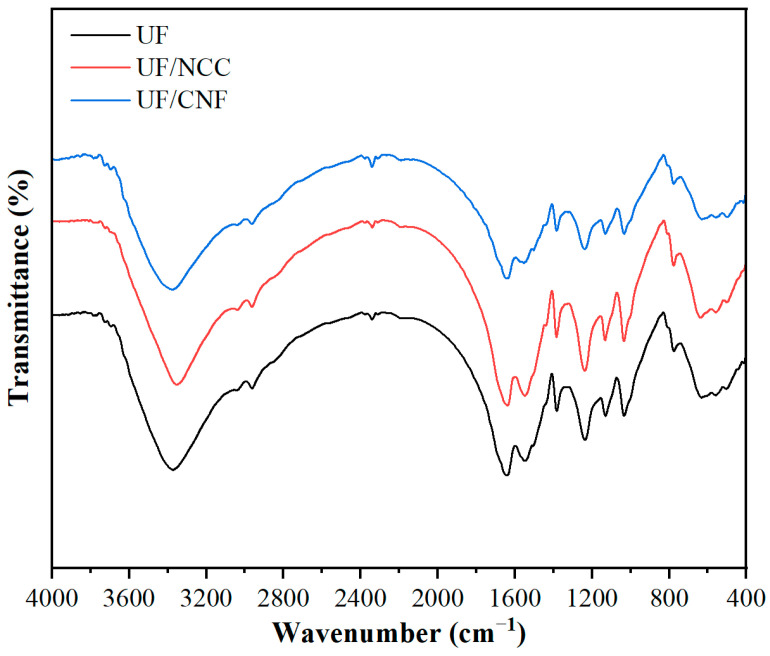
Infrared spectra of NCC- and CNF-modified UF resins.

Figure 2 shows the infrared spectra of NCC- and CNF-modified UF resins. In the region of 3200–3400 cm^−1^, unmodified UF shows broader -OH stretching vibration absorption peaks, indicating more hydrogen bonding and hydration in its molecules. Both NCC- and CNF-modified UF show a narrowing trend of this peak, indicating that the introduction of NCC and CNF changed the hydrogen bonding network in the resin. Notably, for CNF-modified UF, the peak narrows more significantly. This implies that the long-chain structure of CNF promotes hydrogen bond formation within the resin, potentially enhancing cross-linking between triazine ring structures. In contrast, the narrowing of NCC-modified UF is milder, indicating a milder effect on hydrogen bonding. The C-N stretching vibrational peak in the resin is significant in the region of 1300–1500 cm^−1^, while the position and intensity of this absorption peak change weakly in NCC- and CNF-modified UF. This indicates that NCC and CNF do not significantly change the C-N bonding properties in the triazine ring of the resin but may affect the cross-linking density of the resin through interaction with resin molecules. In the C-O-C region (1000–1150 cm^−1^), the position and intensity of the absorption peaks of NCC-modified UF are relatively stable, indicating that NCC enhances the microstructural regularity of the resin by increasing the crystallinity of the resin, while CNF-modified UF, on the other hand, shows stronger absorption, indicating that CNF, through its unique long-chain structure and surface hydroxyl groups, has a stronger cross-linking effect on the UF resin, thus increasing the overall cross-linking density of the resin.

Overall, the modification effects of NCC and CNF on UF resins are significantly different. NCC improves the microstructure of the resins mainly through an enhancement of the crystallinity and hydrogen bonding of the resins. CNF, on the other hand, promotes the cross-linking density of the resin through its long-chain structure and abundant surface hydroxyl groups. The mechanisms of action of the two are different, and CNF-modified UF shows a more significant structural modification.

### 3.2. XRD Analysis

**Figure 3:**
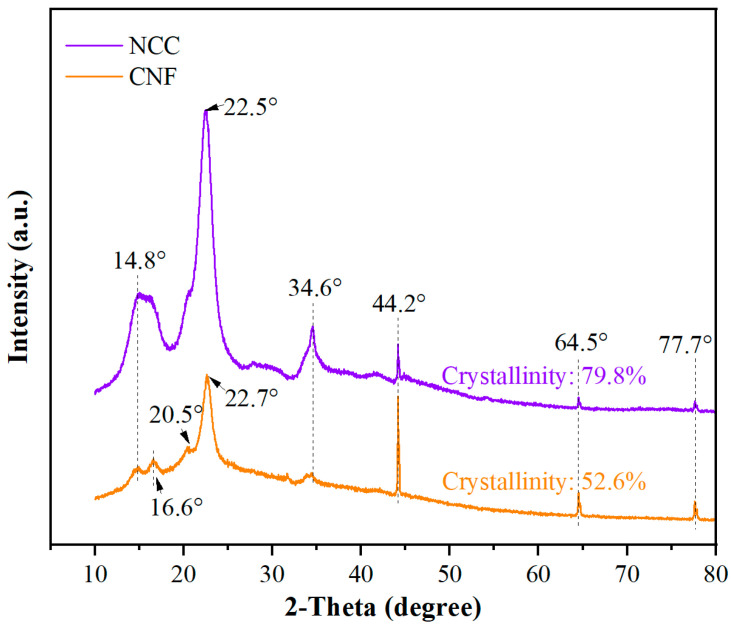
XRD plots of NCC and CNF.

The XRD patterns of NCC and CNF are illustrated in Figure 3. The XRD pattern of NCC shows obvious crystallization peaks, especially at the position of 22.5°, and the peaks are sharp, indicating a high degree of crystallinity. This peak is a typical crystalline structural feature of NCC, reflecting its highly ordered crystal arrangement. In contrast, the XRD pattern of CNF is relatively broad, with major diffraction peaks appearing at 16.6° and 20.5°, indicating a lower degree of crystallinity. CNF exhibits a more disordered structure with a higher specific surface area and poorer crystallinity, a characteristic that makes CNF excellent in terms of dispersibility.

**Figure 4:**
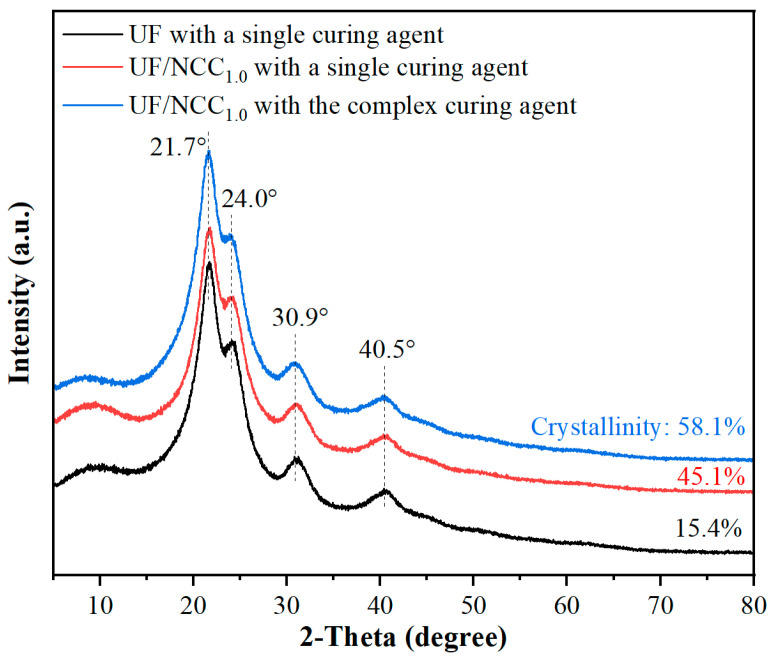
XRD patterns of NCC-modified UF adhesives under single and complex curing agent conditions.

Figure 4 demonstrates the XRD patterns of NCC-modified UF adhesives under single and complex curing agent conditions. The diffraction peaks of the unmodified UF resin were broader and lower in intensity, indicating poor crystallinity. The addition of NCC significantly changed the crystalline structure of the resin under single curing agent conditions. The intensities of the diffraction peaks increased and became sharper at 21.7° and 24.0°, indicating that the crystallinity of the resin was enhanced by the addition of NCC, which, as a kind of nanofibrillar cellulose, provided more cross-linking points and promoted the structural ordering of the UF resin. Under the complex curing agent condition, the diffraction peaks became clearer and further increased in intensity at 21.7° and 24.0°. In addition, new fine peaks appeared near 30.9° and 40.5°, indicating that the complex curing agent helped to improve the dispersion of NCC and promoted the homogeneity of the cross-linking reaction.

**Figure 5:**
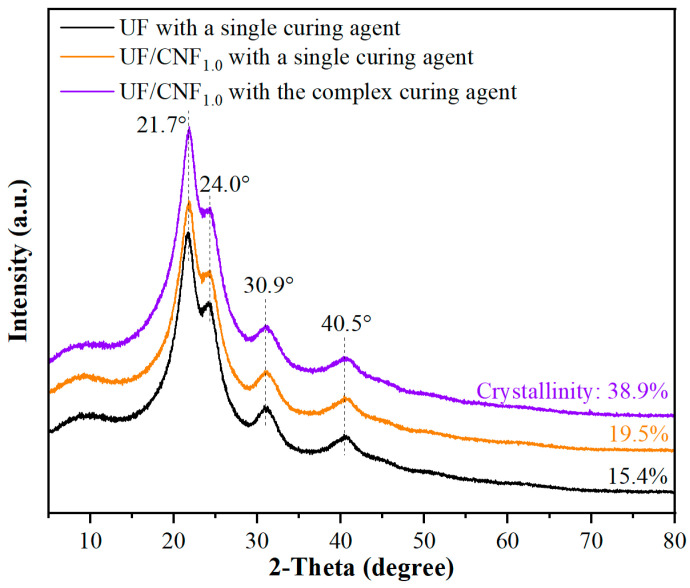
XRD patterns of CNF-modified UF adhesives with single and complex curing agents.

Figure 5 demonstrates the XRD patterns of CNF-modified UF adhesives under single and complex curing agent conditions. Under single curing agent conditions, the addition of CNF significantly changed the crystalline structure of the resin. The diffraction peaks appeared at about 21.7° and 24.0°, and the addition of CNF increased the intensity of these peaks. This suggests that the addition of CNF promotes the crystallization process of the resin and improves the crystallinity and structural ordering of the resin. Under the conditions of the complex curing agent, the diffraction peaks became sharper at 21.7° and 24.0° and the intensity was further enhanced. Especially at 30.9° and 40.5°, more fine diffraction peaks also appeared, indicating that the complex curing agent not only promoted the homogeneous dispersion of CNF but also enhanced the homogeneity of the cross-linking reaction, which further improved the crystallinity and structural stability of the resin.

A comparison of the results in Figure 4 and Figure 5 shows that a single curing agent is able to provide a basic cross-linking reaction but its effect is limited, especially in terms of enhancing the crystallinity and cross-linking uniformity of the resin. The lower crystallinity of the resin and the looser cross-linking between the molecular chains resulted in a limited enhancement of the resin properties. In contrast, complex curing agents provide more cross-linking sites and significantly enhance the cross-linking density of the resin, resulting in sharper and stronger diffraction peaks. In addition, complex curing agents promote uniform cross-linking of the resin, which improves the crystallinity and overall performance of the resin.

The use of NCC and CNF has a significant effect on the crystalline properties of UF adhesives. NCC has a higher degree of crystallinity, which can provide more cross-linking points in the resin and enhance the mechanical strength and water resistance of the resin. Its crystalline structure is more orderly, and the XRD pattern shows strong and sharp diffraction peaks. However, due to the poor dispersibility of NCC, its excessive use may lead to a decrease in the homogeneity of the resin system, thus affecting the performance. In contrast, CNF has a higher specific surface area and better dispersibility, and its fibrous structure enables it to form a more homogeneous cross-linked network in the resin. Despite its relatively low crystallinity, CNF is able to improve the bonding strength and water resistance by enhancing the overall dispersion and structural stability of the resin.

In summary, there is a significant difference in the enhancement of resin properties between single curing agents and complex curing agents, with complex curing agents being able to more effectively enhance the structural stability and crystallinity of the resin. The roles of NCC and CNF in UF resins are distinctive, with NCC providing higher crystallinity and CNF improving the overall properties of the resin through better dispersion. The combination of the complex curing agent and CNF significantly promotes a more homogeneous cross-linking reaction in the resin, which further improves the resin properties. This synergistic effect not only enhances the crystallinity and structural stability of the resin but also improves the overall performance of the resin by improving the dispersion of the nanocellulose.

### 3.3. Curing Performance Analysis

**Figure 6:**
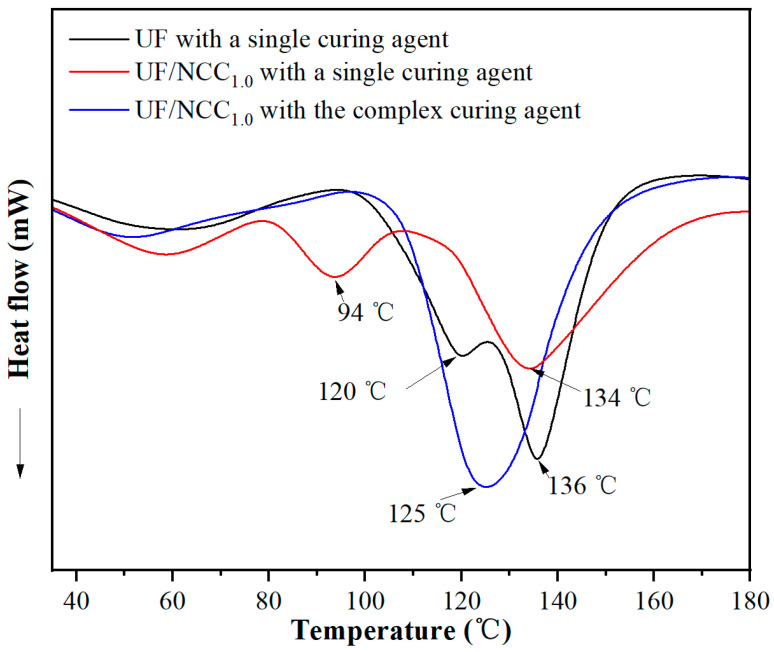
DSC test results of NCC-modified UF adhesives under single and complex curing agent conditions.

Figure 6 shows the DSC test results of NCC-modified UF adhesives under single and complex curing agent conditions. Blank UF showed two exothermic peaks at 120 °C and 136 °C in the presence of a single curing agent, indicating that the curing process of the UF resin consists of two stages, where 120 °C corresponds to the pre-crosslinking reaction of hydroxymethyl groups and 136 °C corresponds to the main exothermic peak of the resin curing (the formation of a stable cross-linked network). The NCC-modified UF also showed two exothermic peaks in the presence of a single curing agent, and the low-temperature exothermic peak decreased from 120 °C to 94 °C compared with that of the blank UF, which may be attributed to the fact that the introduction of NCC provided more active sites and promoted the resin to undergo a partial cross-linking reaction at a lower temperature. In addition, the main curing peak temperature decreased from 136 °C to 134 °C, indicating that the addition of NCC accelerated the curing reaction of adhesive. The NCC-modified UF showed only a single exothermic peak at 125 °C under the complex curing agent condition, implying a significant change in the curing reaction. Compared with the single curing agent system, the decrease in the temperature of the main exothermic peak indicates that the complex curing agent can catalyze the curing reaction more efficiently and reduce the multi-stage cross-linking process, resulting in a more concentrated and homogeneous reaction. In addition, the low-temperature exothermic peak disappeared, indicating that the complex curing agent optimized the influence of NCC on the curing process and improved the stability of the curing system.

**Figure 7:**
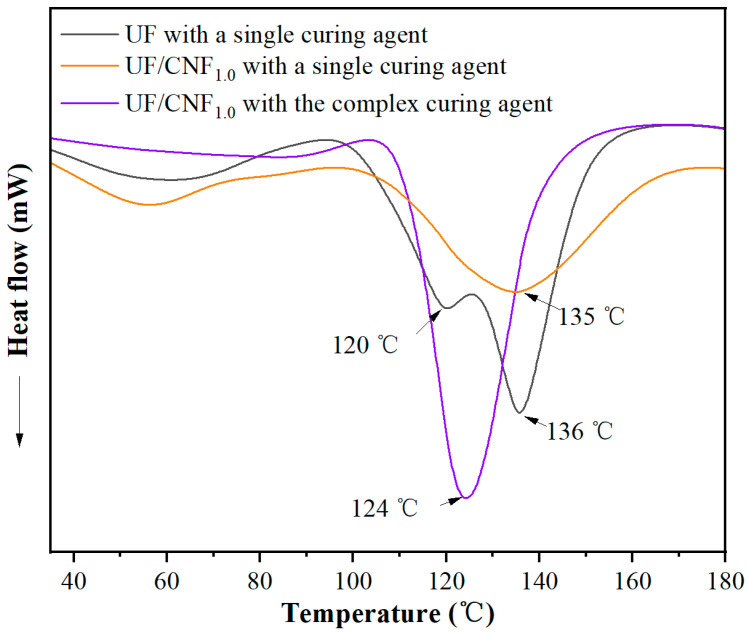
DSC test results of CNF-modified UF adhesives with single and complex curing agents.

Figure 7 shows the DSC test results of CNF-modified UF adhesives under single and complex curing agent conditions. CNF-modified UF with single curing agent only showed a single exothermic peak at 135 °C, indicating that the addition of CNF made the curing process more homogeneous and eliminated the low-temperature exothermic phase. CNF-modified UF with the complex curing agent also showed a single exothermic peak at 124 °C, indicating that the introduction of complex curing agent further optimized the curing process and made the curing phase more concentrated. Compared with the single curing agent, the curing temperature was significantly reduced, indicating that the complex curing agent improved the reactivity of the curing system, which enabled the resin to complete the cross-linking curing at a lower temperature. The appearance of a single exothermic peak indicates that the complex curing agent enhances the promotion effect of CNF on the curing reaction so that the whole curing process tends to be completed in one step, which improves the cross-linking density and reduces the inhomogeneity of the system.

In summary, the addition of NCC may lead to enhanced early cross-linking of the resin, and CNF may make it easier to change the curing process of UF resin from a two-stage to a single-stage process, improve the homogeneity of the curing process, and make the curing reaction more concentrated. Compared with a single curing agent, the use of a complex curing agent can improve the catalytic efficiency, further optimize the curing process, make the curing reaction more rapid and homogeneous, promote rapid cross-linking of the resin at lower temperatures, and improve the stability and overall performance of the curing system. In addition, the synergistic effects of CNF and the complex curing agent on the curing behavior of UF resin are significant, which are important in optimizing the performance of the adhesive.

### 3.4. Thermal Stability Analysis

**Figure 8:**
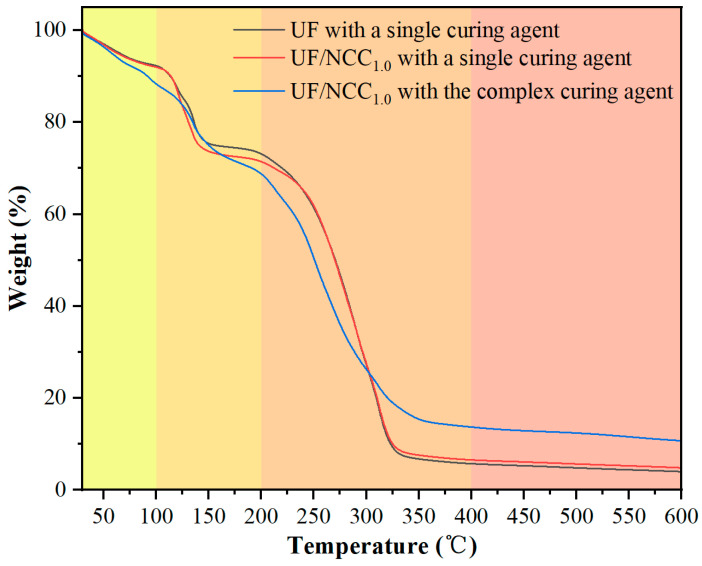
TG test results of NCC-modified UF adhesives under single and complex curing agent conditions.

Figure 8 shows the TG test results of NCC-modified UF adhesives under single and complex curing agent conditions. The weights of blank UF were 92.3%, 73.1%, 5.7%, and 4.0% at 100 °C, 200 °C, 400 °C, and 600 °C when it was exposed to a single curing agent, which indicated that it was thermally stable until 200 °C, but rapidly degraded after 400 °C and had poor heat resistance. When NCC-modified UF was exposed to a single curing agent, the weight decreased at 100 °C and 200 °C (91.4% and 70.8%), indicating that NCC might have accelerated part of the low-temperature degradation; however, the weight (6.0% and 4.2%) was slightly higher than that of the blank UF at 400 °C and 600 °C, which indicated that NCC promoted carbonation at the high-temperature stage to improve the heat resistance. When the NCC-modified UF was exposed to the complex curing agent, the weight decreased to 88.4% and 68.8% at 100 °C and 200 °C, indicating that the complex curing agent might have changed the cross-linking structure, which made the resin more easily degraded at the low-temperature stage; however, the weight increased dramatically to 13.7% and 10.7% at 400 °C and 600 °C, which indicated that the complex curing agent enhanced the high-temperature carbonization and improved the heat resistance.

**Figure 9:**
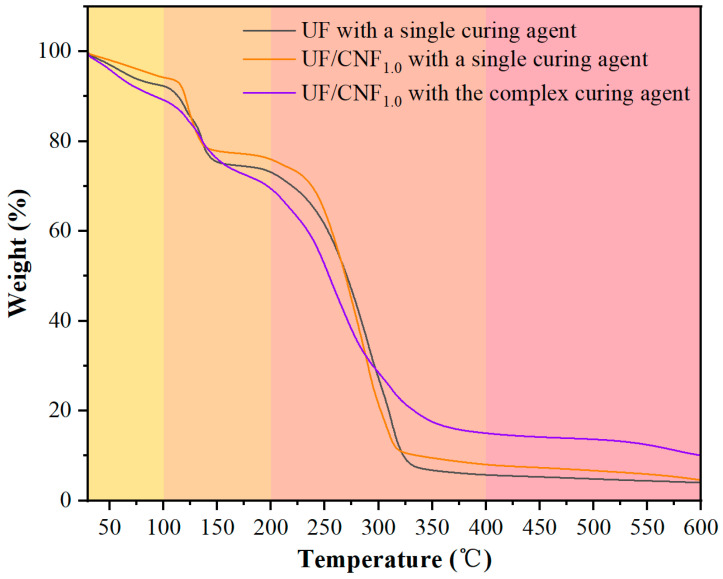
TG test results of CNF-modified UF adhesives with single and complex curing agents.

Figure 9 shows the TG test results of CNF-modified UF adhesives under single and complex curing agent conditions. CNF-modified UF has slightly higher weight at 100 °C and 200 °C than the blank UF (94.2% and 76.0%) when exposed to a single curing agent, which indicates that CNF has a positive effect on the low-temperature stability. At low temperatures, the formation and breaking of hydrogen bonds are relatively slow. The hydrogen bond interaction between NCC and the resin matrix may compete with the cross-linking effect of the resin itself, interfering with the formation of the normal cross-linking network of the resin matrix. The weights at 400 °C and 600 °C (8.0% and 4.5%) were higher than those of blank UF, indicating that the CNF modification improved the overall heat resistance. When CNF-modified UF was exposed to the complex curing agent, the weights at 100 °C and 200 °C (89.2% and 69.4%) were similar to those of the NCC-modified resin, but at 400 °C and 600 °C, the weights were 15.0% and 11.3%, which were higher than those of the NCC-modified system, suggesting that CNF promotes high-temperature carbonization more strongly.

Comparing the results of the above analysis, it can be seen that the differences and reasons for the effects of NCC and CNF on the thermal stability of UF resin may be mainly reflected in two aspects. In the low-temperature stage (≤200 °C), the addition of NCC may destroy part of the cross-linking network of the resin matrix and lower the short-chain degradation temperature, leading to a decrease in the amount of residual carbon at 200 °C, whereas CNF, due to its stronger hydrogen bonding, can improve the thermal stability of the resin and delay its low-temperature decomposition. In the high-temperature stage (≥400 °C), both NCC (13.7% residual carbon) and CNF (15.0% residual carbon) can promote carbonization and increase the residual carbon content, but CNF is more capable of carbonization, which indicates its better stability at high temperature. In addition, the complex curing agent can further enhance the high-temperature carbonization and increase the residual carbon rate. Taken together, the CNF-modified UF has better overall thermal stability than the NCC-modified system, especially in the low- and high-temperature stages. The addition of the complex curing agent can further increase the residual carbon content at high temperatures, indicating that it enhances the carbonization of the resin. In practical applications, if the focus is on low-temperature stability, the CNF modification can be preferred; if the focus is on high-temperature heat resistance, the synergistic use of complex curing agents is more critical.

### 3.5. SEM Analysis

**Figure 10:**
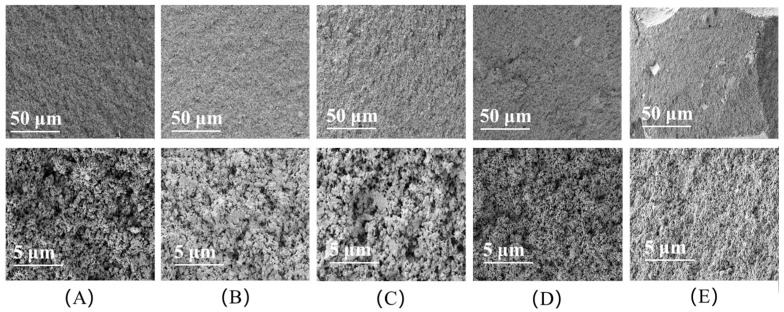
Apparent morphology of the cured products of modified UF adhesives. (**A**) The cross-section SEM image of the blank UF cured product, (**B**) the cross-section SEM image of the UF cured product modified with NCC by a single curing agent, (**C**) the cross-section SEM image of the UF cured product modified with NCC by the complex curing agent, (**D**) the cross-section SEM image of the UF cured product modified with CNF by the single curing agent, and (**E**) the cross-section SEM image of the UF cured product modified with CNF by the complex curing agent.

Figure 10 shows the surface morphology of the cured products of modified UF adhesives. The surface of the cured product of the blank UF resin without the addition of curing agent was rough and there was a certain degree of delamination or microscopic cracks (A). This indicated that a close three-dimensional crosslinked network was not formed during the curing process, and the intermolecular interactions were weak, resulting in the surface structure of the cured product not being dense enough, and the mechanical properties might be lower. The molecular chains of the resin failed to cross-link sufficiently, resulting in it still showing high micro-uniformity after curing.

NCC was poorly dispersed, and with a single curing agent, some aggregated granular structures were formed with a rougher surface (B). With the complex curing agent, NCC was more uniformly distributed, the granular structure was reduced, and the surface was more regular (C). This indicated that the complex curing agent enhanced the distribution uniformity of NCC in the UF resin by improving its dispersion, which further promoted the cross-linking and strengthening of the resin.

CNF has longer molecular chains, a higher specific surface area, and stronger dispersing ability. With a single curing agent, the surface of CNF-modified UF showed a more uniform distribution without obvious granularity (D), which indicated that CNF was better dispersed in the resin matrix and formed a denser structure. The complex curing agent further optimized the dispersion of CNF, resulting in a more uniform distribution of fibers and a smoother overall surface (E), showing a more desirable curing effect.

In summary, CNF shows better dispersibility and homogeneity due to its longer molecular chain and stronger dispersing ability, especially under the effect of the compound curing agent, and the resin structure is more dense. NCC, on the other hand, is easy to aggregate due to its strong crystallinity, which affects its dispersibility and the cross-linking effect on the resin. The complex curing agent has significantly improved the dispersibility and cross-linking effect of both NCC and CNF, especially CNF, and the effect of the complex curing agent has led to a more homogeneous distribution in the cured product, which further improves the overall performance of the resin.

### 3.6. Analysis of the Gluing Properties

**Figure 11:**
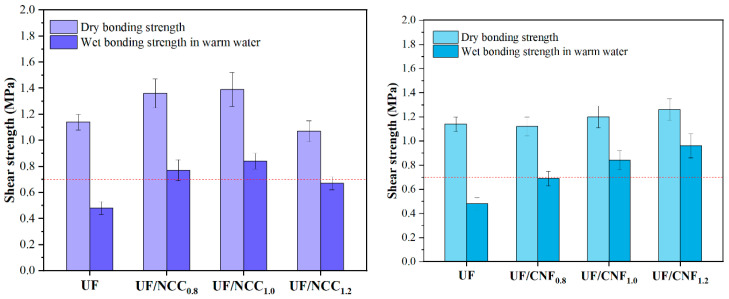
Test results for the adhesive strength of UF resins modified with NCC and CNF in the presence of a single curing agent.

Figure 11 shows the test results of the gluing strength of UF resins modified with NCC and CNF in the presence of a single curing agent. Under the single curing condition of ammonium chloride, both NCC and CNF significantly enhanced the gluing performance of UF, but the effect of CNF was more significant. Before modification, the dry bonding strength of UF was 1.14 MPa and the water-resistant bonding strength was 0.48 MPa. With the addition of 0.8% NCC, the dry bonding strength was increased to 1.36 MPa and the water-resistant bonding strength was increased to 0.77 MPa. By further increasing the amount of NCC to 1.0%, the dry bonding strength was increased to 1.39 MPa and the water-resistant bonding strength was increased to 0.84 MPa, which exhibited optimal gluing performance. However, when the amount of NCC added was increased to 1.2%, the dry bonding strength decreased to 1.07 MPa, and the water-resistant bonding strength was 0.67 MPa. This indicated that the addition of NCC significantly enhanced the bonding strength of UF, especially the water-resistant property, but an excessive amount of NCC might lead to the deterioration of dispersion in the adhesive system, which would in turn affect the homogeneity of the cross-linking reaction.

In comparison, CNF showed a better modification effect under the same conditions. When 0.8% CNF was added, the dry bonding strength was 1.12 MPa, and the water-resistant bonding strength was increased to 0.69 MPa; when 1.0% CNF was added, the dry bonding strength was increased to 1.20 MPa, and the water-resistant bonding strength was further increased to 0.84 MPa; and when 1.2% CNF was added, the dry bonding strength was 1.26 MPa, and the water-resistant bonding strength was increased to 0.96 MPa. This is because the high specific surface area and good dispersibility of CNF enable it to form a closer cross-linking network with UF, thus significantly improving the cohesion and water resistance of the adhesive.

**Figure 12:**
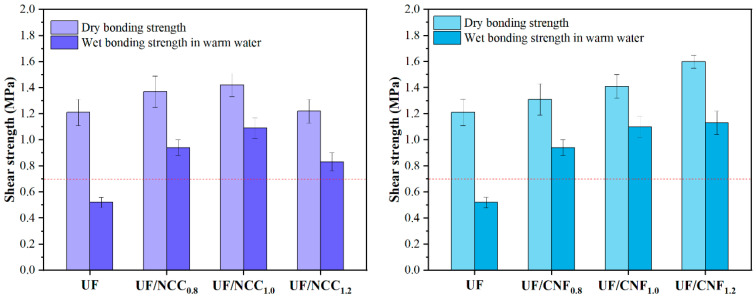
Test results for the adhesive strength of UF resins modified with NCC and CNF in the presence of the complex curing agent.

Figure 12 shows the test results of the NCC- and CNF-modified UF resin gluing strength with the complex curing agent. The effects of the NCC and CNF modifications were further enhanced under complex curing conditions, especially for CNF. Before modification, the dry bonding strength of the UF resin was 1.21 MPa, and the water-resistant bonding strength was 0.52 MPa. With the addition of 0.8% NCC, the dry bonding strength was increased to 1.37 MPa, and the water-resistant bonding strength was increased to 0.94 MPa; with the addition of 1.0% NCC, the dry bonding strength was reached to 1.42 MPa, and the water-resistant bonding strength was increased to 1.09 MPa; and with the addition of 1.2% NCC, the dry bonding strength was 1.22 MPa, and the water-resistant bonding strength was 0.87 MPa. This indicated that the complex curing agent significantly enhanced the performance of NCC-modified UF resin, especially the water-resistant adhesive strength.

CNF showed a more significant modification effect under complex curing conditions. With the addition of 0.8% CNF, the dry bonding strength was increased to 1.31 MPa, and the water-resistant bonding strength was increased to 0.94 MPa; with the addition of 1.0% CNF, the dry bonding strength reached 1.41 MPa, and the water-resistant bonding strength was significantly increased to 1.10 MPa; and with the addition of 1.2% CNF, the dry bonding strength was 1.60 MPa, and the water-resistant bonding strength reached 1.13 MPa. These results indicated that the complex curing agent further promoted the cross-linking reaction between CNF and the UF resin by providing more cross-linking points to form a denser network structure, which significantly enhanced the cohesion and water resistance of the adhesive.

The structural properties of NCC and CNF determine the gluing performance of modified UF adhesives. NCC is usually in a granular or rod-like form with high crystallinity and stability, which can provide more cross-linking points and thus improve the cohesion and water resistance of the adhesive. However, its poor dispersibility may lead to insufficient homogeneity of the adhesive system, which in turn affects the final performance. In contrast, CNF is in the form of flexible fibers with a high specific surface area and good dispersibility, which can be more uniformly distributed in the UF resin to form a denser crosslinked network. This not only improves the cohesion of the adhesive but also significantly improves its water resistance.

In addition, the composition of the curing agent also has a more significant effect on the performance of modified UF adhesives. Under the condition of single curing agent, both NCC and CNF can significantly improve the performance of the UF resin, but the effect of CNF is more significant. The complex curing agent further enhances the modification effects of NCC and CNF; especially, the performance enhancement of the CNF-modified UF resin is more significant. This indicates that there is a good synergistic effect between the complex curing agent and CNF, which can further optimize the comprehensive performance of the adhesive.

### 3.7. Analysis of Formaldehyde Release

**Figure 13:**
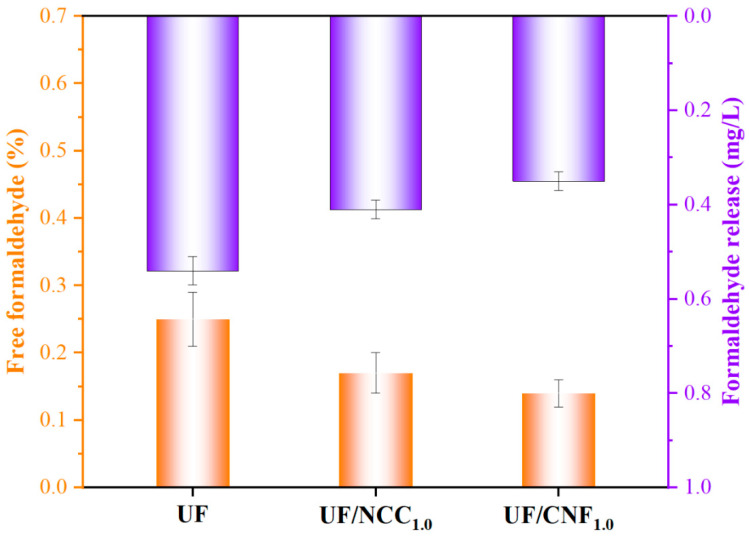
Free formaldehyde content of NCC- and CNF-modified UF adhesives and their test results for formaldehyde emissions from the plywood produced.

Figure 13 shows the free formaldehyde content of blank UF, UF/NCC1.0 and UF/CNF1.0 adhesives and their test results for formaldehyde emissions from the plywood produced. The incorporation of nanocellulose (NCC and CNF) significantly reduced the free formaldehyde content in UF resins. Specifically, the free formaldehyde content of the unmodified UF resin was 0.25%, whereas the free formaldehyde content of the UF resin (UF/NCC1.0) modified by adding 1.0% NCC was reduced to 0.17%, and the free formaldehyde content of the UF resin (UF/CNF1.0) modified by adding 1.0% CNF was further reduced to 0.14%. This indicates that the addition of nanocellulose material can effectively reduce the content of free formaldehyde in the UF resin.

Formaldehyde emissions from plywood also decreased significantly with the addition of nanocellulose. The formaldehyde emission from plywood prepared with the unmodified UF resin was 0.54 mg/L, while the formaldehyde emissions from plywood prepared with UF/NCC1.0 and UF/CNF1.0 were reduced to 0.41 mg/L and 0.35 mg/L. This result indicates that the nanocellulose material not only reduces the content of free formaldehyde at the resin synthesis stage but also further reduces the formaldehyde release from the final product.

Although both NCC and CNF could significantly reduce the free formaldehyde content in the UF resin and formaldehyde release from plywood, the modification effect of CNF was more significant. This difference may be closely related to the structural properties and their interaction mechanism with the UF resin. The insufficient dispersion of NCC may lead to a decrease in the uniformity of the adhesive system and the unevenness of the cross-linking network, which in turn affects the fixation of formaldehyde. CNF’s high specific surface area and good dispersibility enable it to undergo a wider cross-linking reaction with hydroxymethyl groups in the UF resin to form a denser and more homogeneous network structure, which not only reduces the free formaldehyde content but also promotes the curing reaction of the resin to further reduce formaldehyde residues.

## 4. Conclusions

This research showed that the incorporation of NCC and CNF significantly affected the physicochemical properties of UF resins. NCC improves the microstructure of the resin by enhancing its crystallinity and hydrogen bonding, which makes the resin more regular and enhances its mechanical strength and water resistance. Meanwhile, CNF improves the dispersion of the resin and promotes the cross-linking density through its long-chain structure and high specific surface area, thus enhancing the resin’s thermal stability, mechanical properties, and water resistance. The use of complex curing agents further optimizes the cross-linking reaction of the resin, resulting in a more homogeneous curing process and improving the crystallinity and stability of the resin. The action mechanisms of NCC and CNF in the resin are different, with the former focusing on enhancing the crystallinity of the resin and the latter enhancing the overall performance of the resin by improving the dispersion. The gluing performance tests showed that the CNF-modified UF adhesives exhibited excellent performance in both dry and water-resistant gluing strengths, and the use of the complex curing agent was able to significantly improve the gluing strength. The formaldehyde release analysis further verified that the incorporation of nanocellulose effectively reduced the free formaldehyde content in the resin, especially CNF, indicating that it could improve the environmental friendliness of the resin. In summary, CNF is more suitable than NCC for the modification of UF resins, especially in improving the structural stability and thermal stability and reducing formaldehyde release from the resins, with more significant advantages.

## Data Availability

The original contributions presented in this study are included in the article. Further inquiries can be directed to the corresponding author.
